# Comparing Sugarbaker versus keyhole mesh technique for open retromuscular parastomal hernia repair: study protocol for a registry-based randomized controlled trial

**DOI:** 10.1186/s13063-022-06207-x

**Published:** 2022-04-04

**Authors:** Benjamin T. Miller, Jonah D. Thomas, Chao Tu, Adele Costanzo, Lucas R. A. Beffa, David M. Krpata, Ajita S. Prabhu, Michael J. Rosen, Clayton C. Petro

**Affiliations:** 1 Department of Surgery, Cleveland Clinic Center for Abdominal Core Health, Cleveland Clinic Foundation, 9500 Euclid Avenue, Cleveland, OH 44195 USA; 2 Department of Surgery, Massachusetts General Hospital, 55 Fruit Street, Boston, MA 02114 USA; 3 Department of Statistics, Cleveland Clinic Foundation, 9500 Euclid Avenue, Cleveland, OH 44195 USA

**Keywords:** Parastomal hernia, Retromuscular, Sugarbaker, Keyhole, Transversus abdominis release, Randomized controlled trial

## Abstract

**Background:**

Parastomal hernia, common after stoma creation, negatively impacts patient quality of life. For patients with a permanent stoma, durable parastomal hernia repair remains a challenge, with few high-quality studies for guidance. An alternative to open retromuscular parastomal hernia repair with retromuscular “keyhole” mesh is the recent Sugarbaker modification. We aim to compare these two techniques in a head-to-head prospective study.

**Methods:**

This is a registry-based randomized controlled trial designed to investigate whether the retromuscular Sugarbaker technique is superior to the retromuscular keyhole technique for parastomal hernia repair. The primary study endpoint is parastomal hernia recurrence at 2 years. Secondary endpoints include hospital length-of-stay, readmission, wound morbidity, mesh-related complications, re-operation, all 30-day morbidity, and patient-reported outcomes, including hernia-related quality of life, stoma-specific quality of life, pain, and decision regret.

**Discussion:**

Based on the post hoc analysis of a recent randomized controlled trial, we hypothesize that the retromuscular Sugarbaker technique will reduce parastomal hernia recurrence by 20% at 2 years compared to the retromuscular keyhole mesh technique. The results of this study may provide evidence-based guidance for surgeons repairing parastomal hernias.

**Trial registration:**

ClinicalTrials.gov NCT03972553. Registered on 3 June 2019

## Administrative information

Note: the numbers in curly brackets in this protocol refer to SPIRIT checklist item numbers. The order of the items has been modified to group similar items (see http://www.equator-network.org/reporting-guidelines/spirit-2013-statement-defining-standard-protocol-items-for-clinical-trials/).

**Table:** 

Title {1}	Comparing Sugarbaker versus keyhole mesh technique for open retromuscular parastomal hernia repair: study protocol for a registry-based randomized controlled trial
Trial registration {2a and 2b}.	ClinicalTrials.gov NCT03972553; June 3, 2019
Protocol version {3}	Version 1.3, June 22, 2020.
Funding {4}	This study is funded by a $20,000 grant from the Central Surgical Association.
Author details {5a}	Benjamin T Miller, MD Department of Surgery, Cleveland Clinic Center for Abdominal Core Health Cleveland Clinic Foundation, Cleveland, Ohio Jonah D. Thomas, MD, MS Department of Surgery, Massachusetts General Hospital Boston, Massachusetts Chao Tu, MS Department of Statistics, Cleveland Clinic Foundation, Cleveland, Ohio Adele Costanzo, RN Department of Surgery, Cleveland Clinic Center for Abdominal Core Health Cleveland Clinic Foundation, Cleveland, Ohio Lucas A Beffa, MD Department of Surgery, Cleveland Clinic Center for Abdominal Core Health Cleveland Clinic Foundation, Cleveland, Ohio David M Krpata, MD Department of Surgery, Cleveland Clinic Center for Abdominal Core Health Cleveland Clinic Foundation, Cleveland, Ohio Ajita S Prabhu, MD Department of Surgery, Cleveland Clinic Center for Abdominal Core Health Cleveland Clinic Foundation, Cleveland, Ohio Michael J Rosen, MD Department of Surgery, Cleveland Clinic Center for Abdominal Core Health Cleveland Clinic Foundation, Cleveland, Ohio Clayton C Petro, MD Department of Surgery, Cleveland Clinic Center for Abdominal Core Health Cleveland Clinic Foundation, Cleveland, Ohio
Name and contact information for the trial sponsor {5b}	Not applicable, no trial sponsor
Role of sponsor {5c}	Not applicable, no trial sponsor.

## Introduction

### Background and rationale {6a}

Parastomal hernia after stoma creation is common, occurring in nearly 50% of patients [1, 2]. Problems associated with parastomal hernias—difficulty fitting stoma appliances, parastomal skin breakdown, pain, and bowel obstructions—negatively impact quality of life and push patients to seek repair [3, 4]. Some 800,000 Americans were thought to be living with a stoma in 2003, and an estimated 120,000 stomas are created by surgeons in the USA every year [5]. Parastomal hernia repair, however, remains a challenge. There are no prospective, high-quality studies to guide surgeons and patients in choosing the safest and most durable repair technique. Furthermore, parastomal hernia recurrence rates are high and vary widely despite the repair technique [6].

Parastomal hernias can be repaired using open and minimally invasive approaches. Minimally invasive repairs are typically reserved for smaller parastomal hernias without a concomitant midline hernia, while an open approach is usually preferred in larger, more complex parastomal hernias. Mesh augmentation is used in over 90% of parastomal hernia repairs and has been shown to reduce parastomal hernia recurrences [6, 7]. However, the best mesh configuration to prevent hernia recurrence is unknown. Several centers have evaluated an open retromuscular approach, typically with transversus abdominis release (TAR) and retromuscular mesh placement [8, 9]. Retromuscular mesh reinforcement allows for wide overlap of the midline and parastomal defects in a well-vascularized space and keeps the mesh outside the peritoneal cavity, away from the viscera. Given the high incidence of concomitant midline defects for these patients, this is our most commonly utilized technique.

There are two standard mesh configurations to accommodate the stoma in an open retromuscular approach: keyhole and Sugarbaker. In the keyhole technique, the stoma is pulled through aligned apertures in the mesh and abdominal wall. Using this approach, parastomal hernia recurrence rates at a single institution were 11% at 13 months [8]. In the Sugarbaker technique, the apertures in the abdominal wall are offset and the bowel is placed over the mesh in the retromuscular space [10]. Recently, Pauli et al. found the parastomal hernia recurrence rate to be 4.5% at 10 months using the retromuscular Sugarbaker technique [11]. Additionally, a post hoc analysis of a large randomized controlled trial (RCT) comparing biologic versus synthetic mesh showed a parastomal hernia recurrence rate of 10% at 2 years for the Sugarbaker approach versus 30% for the keyhole approach in 108 patients (*p* = 0.07) (unpublished data). These promising results have prompted the urgent need to perform a well-designed RCT evaluating the outcomes of the two most common parastomal hernia repair approaches at our high-volume center.

### Objectives {7}

We aim to compare clinical and patient-reported outcomes between retromuscular synthetic mesh repairs using the keyhole versus Sugarbaker approach. This will be the first RCT to compare outcomes in open parastomal hernia repairs, giving surgeons critical information to appropriately care for these complex patients.

### Trial design {8}

This is a single-center, parallel-group, superiority, registry-based, prospective RCT. Study data will be captured in the Abdominal Core Health Quality Collaborative (ACHQC) registry and supplemented with Research Electronic Data Capture (REDCap®) when necessary.

## Methods: participants, interventions, and outcomes

### Study setting {9}

This study will be performed at a single site, the Cleveland Clinic Center for Abdominal Core Health, part of the Cleveland Clinic Foundation in Cleveland, Ohio.

### Eligibility criteria {10}

Inclusion criteria:

- Adults with a parastomal hernia who are candidates for an open retromuscular parastomal repair with a permanent stoma.

Exclusion criteria:

- Two or more stomas preoperatively
- Insufficient bowel length for either repair technique as determined by the surgeon intraoperatively.

### Who will take informed consent? {26a}

Informed consent from potential trial participants will be obtained by the study investigator, co-investigators, or research personnel during the preoperative visit.

### Additional consent provisions for collection and use of participant data and biological specimens {26b}

Not applicable, no additional consent provisions needed.

### Interventions

#### Explanation for the choice of comparators {6b}

At our high volume center, keyhole parastomal hernia repair is a popular technique for complex parastomal hernia repairs, but it has a fairly high long-term hernia recurrence rate. In several small single-center series, the open retromuscular Sugarbaker approach has promising results. However, these two techniques have never been compared in a prospective head-to-head trial.

#### Intervention description {11a}

All parastomal hernia repairs will be performed by six fellowship-trained surgeons with experience in abdominal wall reconstruction and gastrointestinal surgery.

Surgical operation:

- Patients will be preoperatively marked for a new stoma site by ostomy nurses.
- All patients will receive preoperative antibiotics per Surgical Care Improvement Project guidelines, in addition to pharmacological and mechanical thromboprophylaxis.
- All patients will undergo midline laparotomy and adhesiolysis.
- Stoma re-siting, stoma revision at the same location, or leaving the stoma in situ will be performed at the surgeon’s discretion and documented appropriately.
- After confirming adequate bowel length for either repair technique, patients will be randomized by study coordinators to the Sugarbaker or keyhole technique using a computer-generated treatment allocation in REDCap®.
- Bilateral retromuscular dissections will be performed.

For the keyhole mesh group:

- If the stoma has been left in situ, the posterior rectus sheath is closed and a lateral or superior slit is made in the mesh to accommodate the bowel. The slit in the mesh is then sewn back together with permanent suture, creating an aperture for the bowel and allowing all elements of the abdominal wall and mesh to align.
- If the stoma has been taken down, the bowel is brought through a cruciate incision in the posterior rectus sheath or contiguous peritoneum. The posterior rectus sheath is then closed. The bowel is then pulled though a cruciate incision in the mesh, just large enough to accommodate it, and finally through the stoma opening in the anterior fascia. The aperture in the mesh may be tightened with permanent suture at the surgeon’s discretion. Apertures in the posterior rectus sheath, mesh, and anterior fascia are all aligned.

For the Sugarbaker group:

- If the stoma has been left in situ, a lateral slit is made in the peritoneum and a V-Y peritoneal advancement flap created to “lateralize” the bowel through the peritoneum.
- If the stoma has been taken down, a cruciate incision is made in the lateral peritoneum to accommodate the bowel.
- The posterior rectus sheath is then closed. The bowel then traverses the retromuscular space—over the mesh—to the stoma aperture, which is medial to the cruciate incision in the peritoneum. The length of retromuscular traverse is left to the surgeon’s discretion and bowel length, but in general, it is encouraged to be as long as possible. The bowel is then brought through the anterior fascia medially so the apertures in the abdominal wall are offset.
- A medium-weight, appropriately sized polypropylene mesh is deployed in the retromuscular space for both repair types.
- Closed-suction drains are placed in the retromuscular space. The retromuscular space is irrigated, and the anterior fascia and skin are closed.
- If the stoma was taken down, it is re-matured.

#### Criteria for discontinuing or modifying allocated interventions {11b}

The primary endpoint will be analyzed via intention-to-treat. When patients experience complications requiring re-operation and conversion of the stoma to the alternative treatment arm, all subsequent adverse events will be attributed to the randomized repair technique.

#### Strategies to improve adherence to interventions {11c}

The research coordinator will not randomize participants until the surgeon confirms adequate bowel length for a Sugarbaker repair intraoperatively.

#### Relevant concomitant care permitted or prohibited during the trial {11d}

Concomitant care is not restricted during this trial. Postoperative care will follow our standard enhanced recovery after surgery protocol [12].

#### Provisions for post-trial care {30}

We have no provisions for ancillary or post-trial care, or for compensation to those who suffer harm from trial participation.

### Outcomes {12}

Primary endpoint:

- Two-year parastomal hernia recurrence assessed by:
  - Physical exam
  - Patient-reported recurrence using the colostomy impact score [13]
  - Computed tomography (CT)

Secondary endpoints:

- Hospital length-of-stay
- Readmission
- Re-operation
- Mesh-related complications
- All 30-day morbidity
- Wound morbidity
  - Surgical site infections (SSI)
  - Surgical site occurrences (SSO)
  - Surgical site occurrences requiring procedural intervention (SSOPI)
    - Reoperation
      - Wound debridement
      - Mesh excision
        - Partial
        - Complete
      - Stoma revision
- Patient-reported outcomes at 30 days (± 15 days), 1 year (± 4 months), and 2 years (± 6 months).
  - Short-form Patient-Reported Outcomes Measure Information System (PROMIS) Pain Intensity Scale [14]
  - Hernia-Related Quality-of-Life Survey (HerQLes) score [15]
  - Colostomy Impact Score [13]
  - Decision Regret Scale [16]

### Participant timeline {13}

**Fig. 1:**
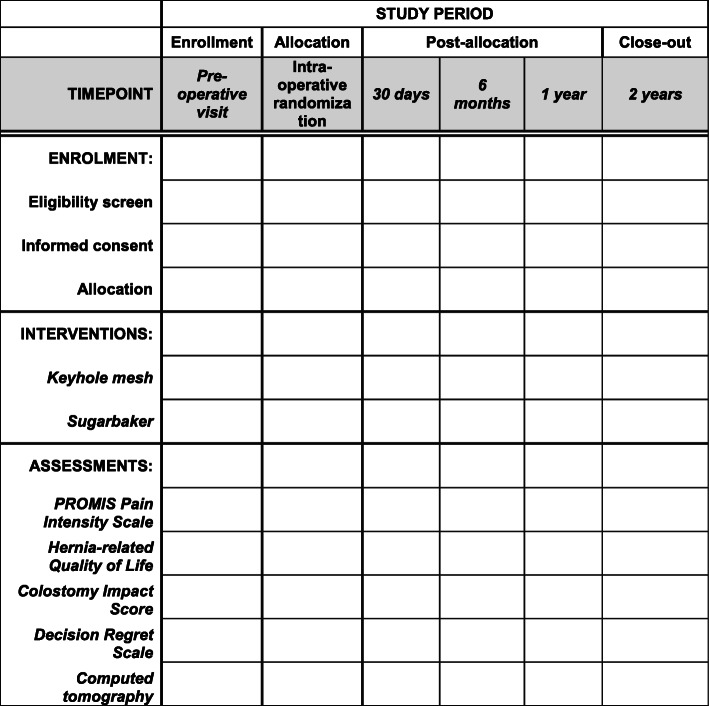
Participant timeline

See participant timeline (Fig. 1).

### Sample size {14}

Based on the post hoc analysis of a large RCT, we predict that the retromuscular Sugarbaker technique will be superior to the keyhole mesh approach and reduce 2-year parastomal hernia recurrence by 20%. With an *α* of 0.05 and power of 80%, we calculated a necessary sample size of 118 patients—59 in each arm to demonstrate this 20% absolute reduction in 2-year parastomal hernia recurrence. Assuming a 20% rate of patients lost to follow-up, an enrollment goal of 142 patients was defined.

### Recruitment {15}

The Cleveland Clinic Center for Abdominal Core Health is a high-volume hernia center, performing approximately 50 parastomal hernia repairs per year. We expect to complete enrollment within 3 years.

## Assignment of interventions: allocation

### Sequence generation {16a}

Intraoperative computer-generated treatment allocation in REDCap® will randomize participants to keyhole versus Sugarbaker technique.

### Concealment mechanism {16b}

Not applicable, the patients are randomized intraoperatively after the surgeon confirms adequate bowel length for either repair technique.

### Implementation {16c}

Participants will be enrolled by the surgeon during the preoperative consultation. The intraoperative allocation sequence will be generated by the research coordinator in REDCap®.

## Assignment of interventions: blinding

### Who will be blinded {17a}

Study participants, those collecting patient-reported outcomes, and 2-year CT scan assessors will be blinded to repair technique.

### Procedure for unblinding if needed {17b}

Participants may be unblinded to their repair technique if they experience complications requiring re-operation.

## Data collection and management

### Plans for assessment and collection of outcomes {18a}

Patient characteristics and operative details will be captured in the ACHQC database, per our practice standard. Patient-reported quality of life at baseline will be collected with the standard ACHQC questionnaire, which includes the short-form PROMIS Pain Intensity Scale [14] and HerQLes, validated for assessing quality-of-life related to abdominal wall function [15]. Preoperative stoma-related quality of life will also be assessed using the validated Colostomy Impact Score questionnaire [13].

Postoperative clinical outcomes, including hospital length-of-stay, readmission, re-operation, mesh-related complications, and wound morbidity, will be captured in the ACHQC registry and in REDCap®. Wound morbidity, including SSIs, SSOs, and SSOPIs, will be reported using standard definitions [17].

Postoperative visits at 30 days, 1 year, and 2 years postoperatively (our practice standard-of-care) will include a physical exam and collection of patient-reported outcomes. Patient-reported outcomes will be assessed using the standard postoperative ACHQC questionnaire, which includes the short-form PROMIS Pain Intensity Scale and HerQLes. Additional postoperative questionnaires will capture Colostomy Impact Scores and Decision Regret Scales, validated for measuring regret after health care decisions [16].

Parastomal hernia recurrence will be evaluated by physical exam, patient-reported outcomes (colostomy impact score), and radiography. Physical exam will identify a parastomal hernia if there is bulging in the vicinity of the stoma. Colostomy impact score will identify a recurrence if patients respond “Yes, I have a small bulge (under 10 cm)” or “Yes, I have a larger bulge (over 10 cm).” CT recurrence will be defined as protrusion of a separate and distinct segment of bowel or other intra-abdominal contents within the musculofascial aperture. Three surgeons, blinded to the operative surgeon and repair technique, will evaluate radiographic recurrences, and at least two of three assessors must agree on radiographic hernia recurrence for it to be documented as such.

Parastomal hernia recurrence reporting will likely be influenced by missing data and by the many possible permutations of physical exam, patient-reported outcomes, and radiographic findings, so a consensus definition of hernia recurrence was established using all possible scenarios [18]. Two additional definitions of hernia recurrence will be included for sensitivity analyses, performed to evaluate hernia recurrence over its ranges by defining the best- and worst-case scenarios.

1. Best-case scenario: hernia recurrence calculated on the most conservative definition of recurrence (maximum specificity), using the algorithm to report the lowest recurrence rate.
2. Worst-case scenario: hernia recurrence calculated on the most inclusive definition of recurrence (maximum sensitivity), using the algorithm to report the highest recurrence rate.

### Plans to promote participant retention and complete follow-up {18b}

This study is funded by a $20,000 grant from the Central Surgical Association. The grant will be used to reimburse participants’ transportation costs with a $25 gift card for completed 1-yearfollow-up and a $75 gift card for completed 2-yearfollow-up.

### Data management {19}

Patient characteristics and operative details will be captured in the ACHQC database, accessed using an assigned username and password. Treatment arm allocation, postoperative complications, CT results, and patient-reported quality of life scores will be collected in REDCap®, a secure electronic database accessed by the investigator and designated study team members using an assigned login and password. Only the principal investigator, research coordinators, and biostatisticians will have access to patient data for routine data quality assessments and data analyses. All electronic records pertaining to the clinical study will be password-protected, and only approved study members listed on the Cleveland Clinic Foundation Institutional Review Board (IRB) protocol will have password access.

### Confidentiality {27}

Anonymity and confidentiality of subjects participating in this study will be maintained. Every effort will be made to maintain the confidentiality of documents that identify the subject by name (e.g., signed informed consents or clinic charts), except when necessary to allow monitoring by the Office of Research Compliance at the Cleveland Clinic or other regulatory authorities.

### Plans for collection, laboratory evaluation, and storage of biological specimens for genetic or molecular analysis in this trial/future use {33}

Not applicable, we will not obtain biological specimens for genetic or molecular analysis.

## Statistical methods

### Statistical methods for primary and secondary outcomes {20a}

Analyses will be done under the normality assumption, if appropriate. Patient characteristics will be summarized overall and by randomized group, and differences will be described as standardized effects. All analyses, using the intent-to-treat population, will be performed with SAS and R software (version 9.4, Cary, NC; version 4.0.0, Vienna, Austria, respectively). Tests will be considered significant at a 5% level.

The primary study endpoint is 2-year composite hernia recurrence rate as a binary outcome (yes or no). Unadjusted and adjusted logistic regression will compare recurrence rates between the two repair techniques at 2 years. The adjusted model will include pre-specified covariates related to baseline disease severity: patient BMI, recurrent parastomal hernia, stoma disposition (stoma re-matured at a new site versus not), and type of stoma. Results, obtained using g-computation, will be presented as relative and absolute risk differences with a 95% confidence interval. Unbalanced follow-up times will be accounted for by using rate ratio and rate difference. As a sensitivity analysis, we will assess the treatment effect on time to hernia recurrence using a Cox proportional hazard model. Results will be presented by hazard ratio and 95% confidence interval, while adjusting for the pre-specified covariates.

The secondary endpoints are not dependent on the primary endpoint. The secondary endpoints are hospital length-of-stay, readmission, wound morbidity (SSI, SSO, or SSOPI), re-operation (stoma necrosis, mesh-related complication, mucocutaneous stoma disruption, and hernia recurrence requiring reoperation), and patient-reported outcomes, including PROMIS scores, HerQLes scores, opioid consumption at 30 days, stoma-relatedquality-of-life, and decision regret scores. Comparisons of categorical endpoints will be performed using chi-squared tests or Fisher’s exact test. Comparisons of continuous endpoints will be performed using two-sample*t*-tests or Wilcoxon rank-sum tests.

Additional analysis, such as mediation analysis, will be performed if the Data Safety and Monitoring Board (DSMB) or the investigators suspect potential dependent endpoints (for example, SSI and 2-year recurrence).

### Interim analyses {21b}

Interim analysis will not be considered in this study.

### Methods for additional analyses (e.g., subgroup analyses) {20b}

No additional subgroup analysis will be considered.

### Methods in analysis to handle protocol non-adherence and any statistical methods to handle missing data {20c}

The primary endpoint will be analyzed via intention-to-treat. When patients experience complications requiring reoperation and conversion of the stoma to the alternative treatment arm, all subsequent adverse events will be attributed to the randomized repair technique. Sensitivity analysis will be performed using a per-protocol methodology.

Missing data is anticipated to reflect a pattern of missing at random. Multiple imputation will be used to impute missing data at baseline only and provide the missing at random assumption holds. Multiple imputation of follow-up measures will not be performed. If non-random missing data patterns are observed, alternate methods, such as pattern mixture models, will be considered.

### Plans to give access to the full protocol, participant-level data, and statistical code {31c}

We do not have plans for granting public access to the full protocol, participant-level dataset, and statistical code.

## Oversight and monitoring

### Composition of the coordinating center and trial steering committee {5d}

The study principal investigator, co-investigators, and research coordinators at the Cleveland Clinic Center for Abdominal Core Health meet weekly to discuss trial enrollment, randomization, and follow-up. The trial investigators capture patient characteristics and operative details in the ACHQC registry, and the research coordinators are responsible for collecting intraoperative randomization and postoperative outcomes in REDCap®.

### Composition of the data monitoring committee, its role, and reporting structure {21a}

The DSMB meets after every 45 patients enrolled in the trial complete 30-dayfollow-up. The DSMB is comprised of three surgeons who are external to the study. For more information on Cleveland Clinic Foundation’s DSMB structure and reporting visit: https://view.officeapps.live.com/op/view.aspx?src=https%3A%2F%2Fwww.lerner.ccf.org%2Fclinical%2Fcru%2Fdocuments%2FDSMB%2520v3.%25203.03.08.doc.

### Adverse event reporting and harms {22}

All adverse events are captured by the research coordinator and reported annually to the IRB and to the DSMB each time it meets. All serious adverse events (e.g., return to the operating room within 30 days or mesh-related complication) are reported to the DSMB immediately.

### Frequency and plans for auditing trial conduct {23}

Research regulatory officers from the Digestive Diseases and Surgical Institute at the Cleveland Clinic Foundation will audit trials to ensure regulatory compliance.

### Plans for communicating important protocol amendments to relevant parties (e.g., trial participants, ethical committees) {25}

Protocol modifications will be added to the study on the Cleveland Clinic IRB website.

## Dissemination plans {31a}

The investigators plan to publish their results in a scientific journal within 6 months of the primary endpoint.

## Discussion

Parastomal hernias represent a unique surgical challenge due to the inherent presence of a trephination, or hernia, in the muscle, which allows for the stoma to traverse from the abdominal cavity to the skin. In addition to well-described high recurrence rates, there are multiple permutations of techniques for repair, few of which have been well studied. This can lead to significant variability in care for these patients, and there is an urgent need to delineate the outcomes of the technical aspects of these operations.

In designing our trial, it was essential to commit to specific techniques in order to maximize the consistency of the operative interventions offered to patients. Common branch points in operative decision-making include location of the stoma (left in situ, revised at same location, re-sited to new location), transfascial suture fixation versus no fixation of mesh, and mesh material (permanent synthetic versus biologic). Through an iterative process, our group reached consensus as to how we would address these technical points in order to maximize the consistency of each technique in our trial. Regarding location of the stoma, we recognized that each patient’s anatomy and current stoma configuration may vary significantly, and we agreed that the most pragmatic way to ensure enrollment and surgeon autonomy was to leave the management of the stoma location to the discretion of the experienced surgeon. For transfascial fixation, based on our own experience with (suspected fixation-related) mesh erosions into the stomata leading to stoma necrosis, bowel obstruction, and bowel perforation requiring reoperation [19], we agreed that avoidance of any mesh fixation was likely to decrease the rate of mesh erosion and have thus changed our standard practice for all patients, including those not enrolled in our trial. While mesh material for contaminated cases has remained controversial, with a common bias towards using biologic mesh, we recently completed a RCT evaluating the efficacy of biologic vs synthetic mesh in contaminated single-stage ventral hernia repairs and found that synthetic mesh reduced the risk of hernia recurrence by 16% at 2 years and was associated with fewer deep SSIs (unpublished data). Therefore, we agreed that permanent synthetic mesh would be the device of choice for our standard practice and accordingly in this trial.

Additionally, the primary endpoint of this trial is parastomal hernia recurrence at 2 years as a composite of physical exam, patient-reported outcomes, and radiography. Parastomal hernia recurrence, though, is notoriously difficult to define. For physical exam, we have used previous investigators’ definition of parastomal hernia as bulging in the stoma vicinity [20, 21]. Patient-reported outcomes are a validated method of detecting ventral hernia recurrences [22], yet no validated questionnaire for parastomal hernia recurrence exists. Nevertheless, we are using Colostomy Impact Score to assess for parastomal hernia recurrence to add data to the primary endpoint. There is likewise no standard definition for parastomal hernia recurrence on CT. Our definition of CT recurrence as a separate and distinct segment of bowel or other intra-abdominal contents (e.g., omentum) within the musculofascial aperture is similar to that of previous authors [20, 21].

An operation’s success is often focused on clinical outcomes, like hernia recurrence. Perhaps, a more meaningful measure is patient satisfaction. Parastomal hernia repair, often done for quality-of-life—not life-threatening—issues, has high morbidity and recurrence rates. But patients may be willing to accept the operation’s morbidity if they are pleased with the repair results. Even hernia recurrence may be preferable to the pouching issues they had before repair. Alternatively, patients without recurrence may be dissatisfied with parastomal hernia repair due to pain or other lifestyle-limiting issues. By adding a decision regret scale, we hope to learn if patients are ultimately satisfied with the outcome of this procedure.

Despite the prevalence of parastomal hernias and the challenging nature of their repair, no prospective, randomized studies have ever compared repair techniques. By comparing two common repair techniques head-to-head, we seek to give surgeons and patients more high-quality data to guide their approach to parastomal hernia repair.

## Trial status

ClinicalTrials.gov Identifier (NCT03972553) June 3, 2019. Trial enrollment began in April 2019, and we expect to complete enrollment by August 2022.

## Abbreviations

TAR: Transversus abdominis release
RCT: Randomized controlled trial
CT: Computed tomography
ACHQC: Abdominal Core Health Quality Collaborative
REDCap®: Research Electronic Data Capture
SSI: Surgical site infections
SSO: Surgical site occurrences
SSOPI: Surgical site occurrences requiring procedural intervention
PROMIS: Patient-Reported Outcomes Measure Information System
HerQLes: Hernia-Related Quality-of-Life Survey
IRB: Institutional Review Board
DSMB: Data Safety and Monitoring Board
